# Production and characterisation of modularly deuterated UBE2D1–Ub conjugate by small angle neutron and X-ray scattering

**DOI:** 10.1007/s00249-022-01620-1

**Published:** 2022-10-26

**Authors:** Zuzanna Pietras, Anthony P. Duff, Vivian Morad, Kathleen Wood, Cy M. Jeffries, Maria Sunnerhagen

**Affiliations:** 1 Department of Physics, Chemistry and Biology, Division of Chemistry, Linköping University, 581 83 Linköping, Sweden; 2 Australian Nuclear Science and Technology Organisation (ANSTO), New Illawarra Road, Lucas Heights, NSW 2234 Australia; 3 European Molecular Biology Laboratory (EMBL) Hamburg Site, 22607 Hamburg, Germany

**Keywords:** Protein conjugation, Protein deuteration, Small angle neutron scattering, Small angle X-ray scattering, Ubiquitination

## Abstract

This structural study exploits the possibility to use modular protein deuteration to facilitate the study of ubiquitin signalling, transfer, and modification. A protein conjugation reaction is used to combine protonated E2 enzyme with deuterated ubiquitin for small angle X-ray and neutron scattering with neutron contrast variation. The combined biomolecules stay as a monodisperse system during data collection in both protonated and deuterated buffers indicating long stability of the E2–Ub conjugate. With multiphase ab initio shape restoration and rigid body modelling, we reconstructed the shape of a E2–Ub-conjugated complex of UBE2D1 linked to ubiquitin via an isopeptide bond. Solution X-ray and neutron scattering data for this E2–Ub conjugate in the absence of E3 jointly indicate an ensemble of open and backbent states, with a preference for the latter in solution. The approach of combining protonated and labelled proteins can be used for solution studies to assess localization and movement of ubiquitin and could be widely applied to modular Ub systems in general.

**Supplementary Information:**

The online version contains supplementary material available at 10.1007/s00249-022-01620-1.

## Introduction

Ubiquitination is a post-translational modification that regulates various cellular processes, including protein degradation, transcription, and cellular signalling (Hershko and Ciechanover 1998). Modularity is an essential feature of the ubiquitination system, allowing both for assembly of varied functional complexes and for sequential transfer of the modifying Ub entity to specific substrate in a highly regulated manner (Zhao et al. 2020). Specifically, in ubiquitination, an 8.6 kDa ubiquitin protein (Ub) is covalently attached to a target protein by an orchestrated multienzyme process that involves a ubiquitin-activating enzyme (E1), a ubiquitin conjugating enzyme (E2) and a ubiquitin ligase (E3). E1 facilitates formation of a thioester bond between the active site cysteine of E2 and the activated C-terminus of Ub, whereas E3 simultaneously binds E2~Ub and substrate to mediate Ub transfer to a lysine residue in the target protein. Molecular knowledge on how ubiquitin is transferred and translocated is key to future development of ubiquitin-based analytical and pharmaceutical tools (Zhao et al. 2020).

In RING-mediated Ub transfer, the Ub–E2 module pair adopts a closed conformation upon binding E3, to prime the transfer of Ub to substrates (Dou et al. 2012; Plechanovová et al. 2012; Pruneda et al. 2012). Previous studies with SAXS have, together with NMR, indicated that in the absence of E3, the E2–Ub module pair adopts various ensembles of states depending on the nature of the E2 (Pruneda et al. 2011). By a combined structural-mutational strategy, we have recently investigated how dynamics in specific residues in the E2 active site could govern ubiquitination by the E3 TRIM21 (Anandapadamanaban et al. 2019). To investigate whether such mutations also influence modular assemblies we would ideally discern the E2–Ub conjugate within its native E3 ligase complex. However, crystallographic analysis is mainly limited to static structures, NMR tools are limited by the protein size and SAXS cannot distinguish parts of the macromolecular complexes.

To better resolve the nature and propensity of the modular E2–Ub ensemble in the absence of E3, we have adopted a SAXS/SANS strategy. The key advantage of SANS is that one can experimentally control the magnitude of neutron scattering contrast by changing the isotopic composition of the sample including both the macromolecules and their solvent. This is achieved by adjusting the ratio between hydrogen (H) and deuterium (D), as neutrons scattered from H have negative scattering length, in contrast to D and other commonly isotopes found in biological macromolecules. Selective deuteration allows for the application of contrast matching or contrast variation techniques to distinguish between labelled protein components in solution (Duff et al. 2015).

Biodeuteration of recombinant proteins is achieved through substitution of H with D at non-exchangeable hydrogen positions. Commonly, production of deuterated proteins utilizing bacterial expression systems, such as *Escherichia coli*, can see significantly reduced yield due to poor cell adaption to the deuterated growth medium. With correct adaptation and the implementation of bioreactors, high yield of expressed protein can be obtained using just one litre of deuterated media and hydrogenated glycerol (Duff et al. 2015).

In this work, we demonstrate the isopeptide conjugation between deuterated Ub and hydrogenated E2 and employ SANS with contrast variation to structurally characterize and develop low-resolution spatial models of the conjugate in solution. This strategy could be used as a tool to probe the structure and disposition of other Ub-conjugated modular systems e.g., in larger multimodular Ub-tagged protein systems. It opens the possibility of solution studies of the ubiquitination pathway, including the production of selectively deuterated polyubiquitin chains (Faggiano et al. 2016) and follow deubiquitinating enzymes by deuterating Ub probes (Borodovsky et al. 2001; Ekkebus et al. 2013; Jong et al. 2017).

## Materials and methods

### Expression and purification of hydrogenated UBE2D1, hE2

The DNA sequence of the human Ubiquitin-conjugating enzyme UBE2D1 with point mutations: S22R, C85K and D87S, was subcloned into the pET28b vector carrying an N-terminal His_6_-tag and a Tobacco Etch Virus (TEV) protease cleavage site. The plasmid DNA was transformed into 50 µl OneShot BL21*(DE3) Star (Invitrogen) cells using heat shock and incubated in 250 µl SOC medium at 37 °C for 1 h. Next 300 µl of culture was transferred to 10 mL of unlabelled ModC1 medium (Duff et al. 2015) using 40 g/L glycerol as carbon source supplemented with 40 µg/mL of kanamycin and 34 µg/mL of chloramphenicol in a 250 mL flask. The cell culture was shaken at 200 rpm at 37 °C until OD_600_ was greater than 0.4, but less than 1.0 (0.4 < OD_600_ < 1), and then it (7.3 mL) was added to four volumes (29 mL) of fresh medium in 2 L flask. After two generation times (3 h), ModC1 was added to take the volume to 102 mL. At OD_600_ = 0.779 (target < 1.0) the 100 mL culture was used to inoculate 900 mL of fresh ModC1 medium in 2L Bioreactor (Real Time Engineering) at 37 °C, continuously aeriated and with pH kept above 6.2 by controlled base feed with 28% ammonium hydroxide. At OD_600_ = 13.8 (target 12 < OD_600_ < 16), the temperature of bioreactor was set to 20 °C and protein expression was started by induction using 0.5 mM isopropyl β-D-thiogalactopyranoside (IPTG). After 19 h of expression, at OD_600_ = 36.2, shortly after exhaustion of the carbon source, as indicated by a small rise in pH, cells were harvested by centrifugation at 4 °C for 20 min at 8000 × *g*, with a final wet mass of 80.4 g. The hydrogenated E2 enzyme is called hE2 through the text.

The cell pellet of hE2 was resuspended in cold Lysis Buffer containing 100 mL Bugbuster (Novagen), 300 mM NaCl, 100 mg lysozyme (QiaExpressionist), 10 mM β-mercaptoethanol (BME), 500 µg DNAse I (Roche) and EDTA-free protease inhibitor cocktail (Roche) and incubated with stirring for 20 min at 4 °C until a viscous solution indicative of cell lysis was achieved. The lysed cells were centrifuged at 20 000 × *g* at 4 °C for 30 min. The cleared supernatant was injected into onto 5 mL HiTrap column (Cytiva) equilibrated in buffer A (50 mM Tris, 300 mM NaCl, 10 mM Imidazole,10 mM BME, pH 8.0) using an Äkta Explorer (Cytiva). The column was washed extensively with buffer A followed by wash buffer B (buffer A plus 20 mM Imidazole, pH 8.0) until a stable UV absorbtion baseline at 280 nm was reached. The protein was eluted in buffer C (buffer A plus 300 mM imidazole, pH 8.0). Fractions containing high protein concentration were mixed with TEV protease to remove the N-terminal polyhistidine-tag and dialyzed overnight at 4 °C against buffer D (50 mM Tris, 150 mM NaCl, 2 mM TCEP, pH 8.0). To remove any remaining His-tagged protein, the post-TEV cleavage sample was injected into HiTrap column equilibrated with buffer A and the flow-thought fractions were collected and further purified by size exclusion chromatography on a HighLoad 16/600 Superdex 75 (Cytiva) column equilibrated in 50 mM Tris, 150 mM NaCl, 2 mM TCEP, pH 8.0.

### Expression and purification of deuterated ubiquitin, dUb

The method used for protein deuteration was the same as for unlabelled protein, except for the use of D_2_O. The DNA sequence encoding human ubiquitin was cloned into pET28b vector with an N-terminal His_6_-tag and a thrombin cleavage site. The plasmid was transformed into OneShot BL21*(DE3) Star (Invitrogen) cells and incubated for 1 h in 250 µL of SOC medium. The culture was added to 10 mL of ModC1 medium containing 50% v/v D_2_O, 40 g/L unlabelled glycerol, supplemented with 40 µg/mL of kanamycin and 34 µg/mL of chloramphenicol and grown at 37 °C until OD_600_ = 0.42 (target 0.4 < OD_600_ < 1). Further adaptation was done by adding the culture to 4 volumes fresh medium containing 100% v/v D_2_O and antibiotics, which results in 90% v/v D_2_O. After two generation times (6 h), fresh media was added to achieve 100 mL, which was grown to OD_600_ = 0.367. The final inoculum was transferred to the bioreactor containing 900 mL of fresh 90% v/v D_2_O ModC1 with 40 g/L of unlabelled glycerol as the sole carbon source and grown in a 2L Bioreactor at 37 °C, continuously aeriated and base feed with 25% ammonium-d4 deuteroxide (Sigma) to keep pH above 6.2 (pD of 6.6). At an OD_600_ = 14, the temperature was decreased to 20 °C and protein expression was induced via the addition of IPTG to 0.5 mM. Protein expression was carried out for 25 h, reaching a final OD_600_ of 27. The cells were harvested shortly after exhaustion of the carbon source, as indicated by a small rise in pH, by centrifugation at 8000 ×*g*, producing a wet-mass cell pellet of 58 g.

The cell pellet of dUb was resuspended in cold lysis buffer containing 100 mL Bugbuster (Novagen), 300 mM NaCl, 100 mg lysozyme (Sigma), 500 µg DNAse I (Roche) and EDTA-free protease inhibitor cocktail (Roche) and incubated with stirring for 20 min at 4 °C. The lysed cells were centrifugated at 20 000 × *g*, 20 min at 4 °C and the soluble fraction was loaded onto a 5 mL HiTrap column (Cytiva) equilibrated in buffer A (50 mM Tris, 300 mM NaCl, 10 mM imidazole, pH 8.0). The column was then washed with buffer A until stable UV absorption baseline at 280 nm was reached, and the protein was eluted with buffer A containing 300 mM imidazole. Fractions containing dUb were further purified on a HighLoad 16/600 Superdex 75 (Cytiva) column equilibrated in 50 mM Tris, 150 mM NaCl, pH 8.0.

The non-exchangeable average deuteration level of dUb was estimated by MALDI-TOF using partial trypsin digestion comparison of unlabelled and labelled samples. This method produced a single matching peptide (IQDKEGIPPDQQR) in the two spectra, and its deuteration level was 79.0%. Whole protein MS was performed on purified hUB and the purified dUb, revealing a whole protein mass ratio of 1.04231, which corresponds to a deuteration level of 77.7%.

### Preparation of hE2–dUb conjugate

The protocol for conjugating dUb and hE2 via an isopeptide bond was adapted from (Plechanovová et al. 2012). Briefly, a 30 mL reaction mixture containing 50 mM Tris, pH 10, 150 mM NaCl, 5 mM MgCl_2_, 0.8 mM TECP, 200 µM dUb-His_6_, 1 µM human E1-His_6_ (Berndsen and Wolberger 2011), 180 µM hE2, 3 mM ATP was incubated for 24 h at 35 °C. To remove unconjugated hE2, the reaction mixture was added to 3 mL of HisLink™ resin (Promega) equilibrated with buffer A with 20 mM Imidazole and incubated for 30 min at 4 °C. Conjugated hE2–dUb-His_6_ was then eluted from the beads using buffer A with the addition of 150 mM imidazole (pH 8.0) and dialysed overnight in buffer A at 20 °C with Thrombin (Sigma). The post-thrombin cleavage sample was passed through a HisLink™ resin to remove any remaining His_6_-tag material, while the flow-through fractions were injected to a HighLoad 16/600 Superdex 75 (Cytiva) column equilibrated with 50 mM Tris, 150 mM NaCl, 0.5 mM TCEP to isolate hE2–dUb. The final purity of the conjugated proteins was assessed by SDS-PAGE stained with InstantBlue (Sigma) and concentrated using Amicon centrifugal filter unit (Millipore). The sample concentration was quantified by UV absorption at 280 nm and calculated using an extinction coefficient of 1.043.

### Small angle scattering data collection and analysis

SANS data were collected on the QUOKKA instrument (Wood et al. 2018) at the Australian Nuclear Science and Technology Organisations. For buffer exchange into D_2_O, a portion of the hE2~dUb sample was run on a Superdex 75 10/300 (Cytiva) column pre-equilibrated in 100% D_2_O buffer (50 mM Tris, 150 mM NaCl, 0.5 mM TCEP, pD 8.4). The samples in H_2_O and D_2_O buffers were concentrated to approximately 4 mg/mL each and the rest of the samples were obtained by mixing buffer exchanged protein solutions at the suitable ratios to achieve 43, 80, 93% D_2_O content. Data were recorded at 10 °C using the instrument parameters reported in Table S1. The resulting 2D isotropic scattering patterns were reduced to 1D-SANS profiles using IGOR Pro with macros adapted to instrumental parameters of QUOKKA (Kline 2006). Scattering data from two sample-detector distances were merged, and profiles of 43, 80, 93% D_2_O buffers were calculated using a linear combination of scattering data from 0 and 100% D_2_O buffers, following the method of (Furlong et al. 2018).

SAXS data were collected on SAXS/WAXS beamline at the Australian Synchrotron (Kirby et al. 2013) with implemented sheath-flow set-up (Kirby et al. 2016) and in-line SEC. The conjugate eluted from a Superdex 75 5/150 Increase column, equilibrated with 20 mM Tris, 150 mM NaCl, 2 mM TCEP, 0.1% sodium azide (pH 7.5) with a flowrate of 0.2 mL/min while coupled to the SAXS beamline capillary. Initial data reduction was performed using the software ScatterBrain (Mudie 2015) and individual frames were further processed with the software CHROMIXS to produce a final background-subtracted scattering profile (Panjkovich and Svergun 2018).

Data processing and analysis were performed using the ATSAS package (Franke et al. 2017). The determination of size parameters was done using PRIMUS (Konarev et al. 2003) using the Guinier approximation (ln*I*(*q*) vs. *q*^2^, *qR*_g_ < 1.3), from which radii of gyration, *R*_g_, and *I*(0) values are extracted. The pair-distance distribution functions, *p*(*r*), were calculated using GNOM software (Svergun 1992) to estimate maximum particle dimension, *D*_max_. To remove the effects of parasitic (near beam-stop) scattering, the *q*-min of the final datasets were selected using the minimum *q*^2^ from the Guinier approximation, calculated using AUTORG in PRIMUS (Franke et al. 2017), while the final *q*-max was estimated for each dataset based on the stability of the *p*(*r*) transform in reciprocal-space determined using GNOM (Svergun 1992). The program MONSA (Petoukhov and Svergun 2006) was used for ab initio shape reconstruction, with the Scattering Length Densities (SLDs) and volume fractions calculated from MULCh (Whitten et al. 2008). Rigid body modelling was performed using SASREFCV (Petoukhov and Svergun 2006) software using the crystal structure, PDB:4AP4 as a starting template (Plechanovová et al. 2012) and refined against SAS data. The calculation of a theoretical solution scattering curves from available crystal structures was done using CRYSOL/CRYSON (Franke et al. 2017) and CorMap was used to evaluate the fits to the experimental data (Franke et al. 2015). The multistate model ensemble was generated using FoXS webserver (Schneidman-Duhovny et al. 2016). The input model was the crystal structure (PDB: 4AP4) with the defined flexible part of C-terminus of Ub (residues 71–76) and a connection between residues G76 in Ub and K85 in E2. The server generated a pool of 10 000 conformations from the starting structure and models with at least 2 states were selected. A detailed summary of the SAS data collection and analysis is available in Table S1. SAS data sets and models are available at SASBDB (Kikhney et al. 2020) under accession code SASDP34.

## Results and discussion

### Production of modularly deuterated ligated sample

**Fig. 1:**
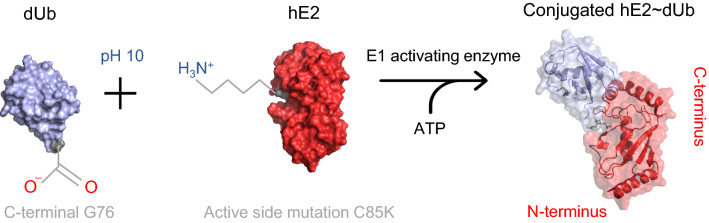
Schematic overview of hE2–dUb conjugate reaction. The formation of isopeptide bond between mutated K85 in the active site of hE2 and dUb. At pH 10, E2 K85 becomes deprotonated to accept dUb from the E1 activating enzyme

In nature, the E2Ub conjugates are linked through an unstable thioester bond between an active site Cysteine (C85) of E2 and the C-terminal Glycine (G76). The catalytic mechanism of ubiquitin transfer cascade is very rapid, thus, to capture specific complexes for structural studies requires the formation of a stable E2–Ub linkage. A point mutation within E2 to Lysine, C58K, allows formation of a more stable isopeptide bond instead (Plechanovová et al. 2011). In this study, the C-terminal G76 of deuterated Ub is ligated to Lysine 85 of hydrogenated E2 (Fig. 1). The reaction is performed in pH over 10 to facilitate deprotonation of the active site Lysine to accept Ub and enabled by incubation with ATP and human E1 enzyme. Additional mutations in E2 include S22R to prevent ubiquitin noncovalent interaction to the backside of E2 (Brzovic et al. 2006) and D87S analogous to the corresponding D133S mutation in UBE2E1 resistant to Ub hydrolysis by TRIM21 (Anandapadamanaban et al. 2019).

**Fig. 2:**
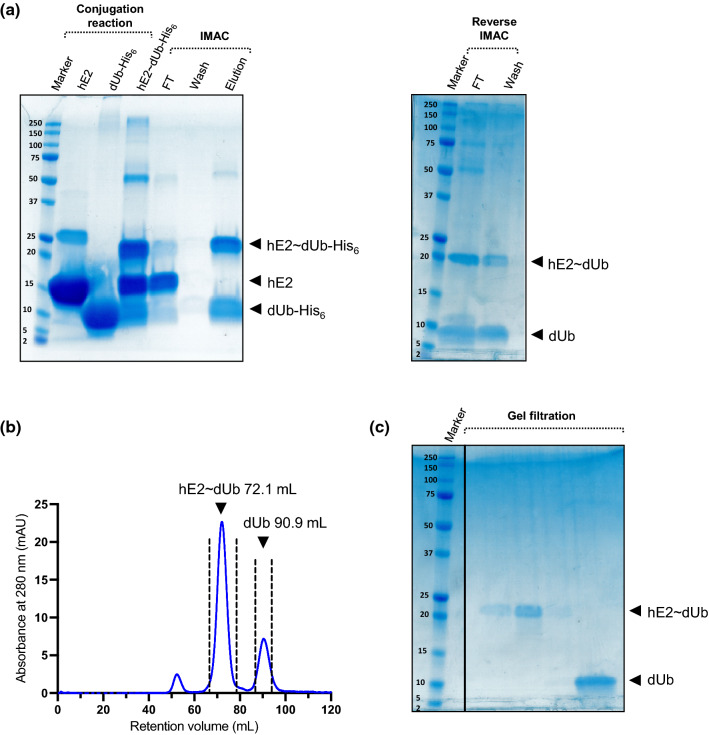
Preparation of a stable hE2~dUb conjugate with an isopeptide bond. a Formation of the conjugate after 24 h reaction at 36 °C and immobilized metal affinity chromatography (IMAC) purification using a HiTrap 5 mL column. After overnight cleavage with TEV protease the sample was purified using reverse IMAC, separating the cleaved material into the flow-through (FT) fraction. We note the appearance of the additional band (~ 25 kDa) on the gel in the purified hE2 line as a dimer. **b** Chromatogram indicating separation of hE2~dUb from unbound dUb. **c** SDS-PAGE showing gel filtration fractions of the peaks

The unique feature of SANS in investigating protein–protein complexes is the possibility to use solution contrast variation by adjusting H_2_O/D_2_O ratios and tailored protein deuteriation levels. In this case, ubiquitin is a great candidate for reactor-based deuteration due to its high expression levels and good solubility. A starter *E. coli* culture of Ub was successfully grown in 50% v/v D_2_O ModC1 media before the final culture was used to inoculate 900 mL of fresh media 90% v/v D_2_O ModC1. The high cell densities were achieved in 2 L bioreactors, and we observed lag-free inoculations. The final yield of dUb from 1 L of 90% v/v D_2_O ModC1 supplemented with hydrogenated glycerol as a carbon source was 64 mg protein. The E2 was expressed in similar manner following three-step adaptation to hydrogenated minimum media. The final yield of hE2 was 92 mg per 1L of ModC1 media, indicating high expression levels and suggesting good protein solubility in high-density cultures. For conjugate preparation samples were concentrated up to 1.271 mM and 0.794 mM, for dUb and hE2, respectively, and SDS-PAGE results showed that both protein samples were of high purity at these concentrations (Fig. 2A). The reaction was carried out for 24 h and the conjugate was further purified to separate unreacted hE2 from the mixture. Under this condition, approximately 50% of the material is ligated as assessed from the elution fraction from IMAC, showed in SDS-PAGE gel in Fig. 2A. The formed His_6_-tagged complex coeluted with His_6_-dUb and both were cleaved with thrombin for His_6_-tag removal before second IMAC (Fig. 2A). Gel filtration was used as a final purification step to separate the conjugate from free Ub, as indicated in the chromatogram in Fig. 2B. Purity of the final sample was evaluated by SDS-PAGE (Fig. 2C) and modularly deuterated conjugate was concentrated to 4 mg/mL for SANS measurements. From 40 mg of purified hE2 and 30 mg of dUb, approximately 4 mg of hE2–dUB conjugate was produced. The main reason for the lower yield was the low efficiency of the thrombin cleavage reaction. In continued studies, this step will be further optimised.

### Characterisation of hE2–dUb conjugate by SAS

**Fig. 3:**
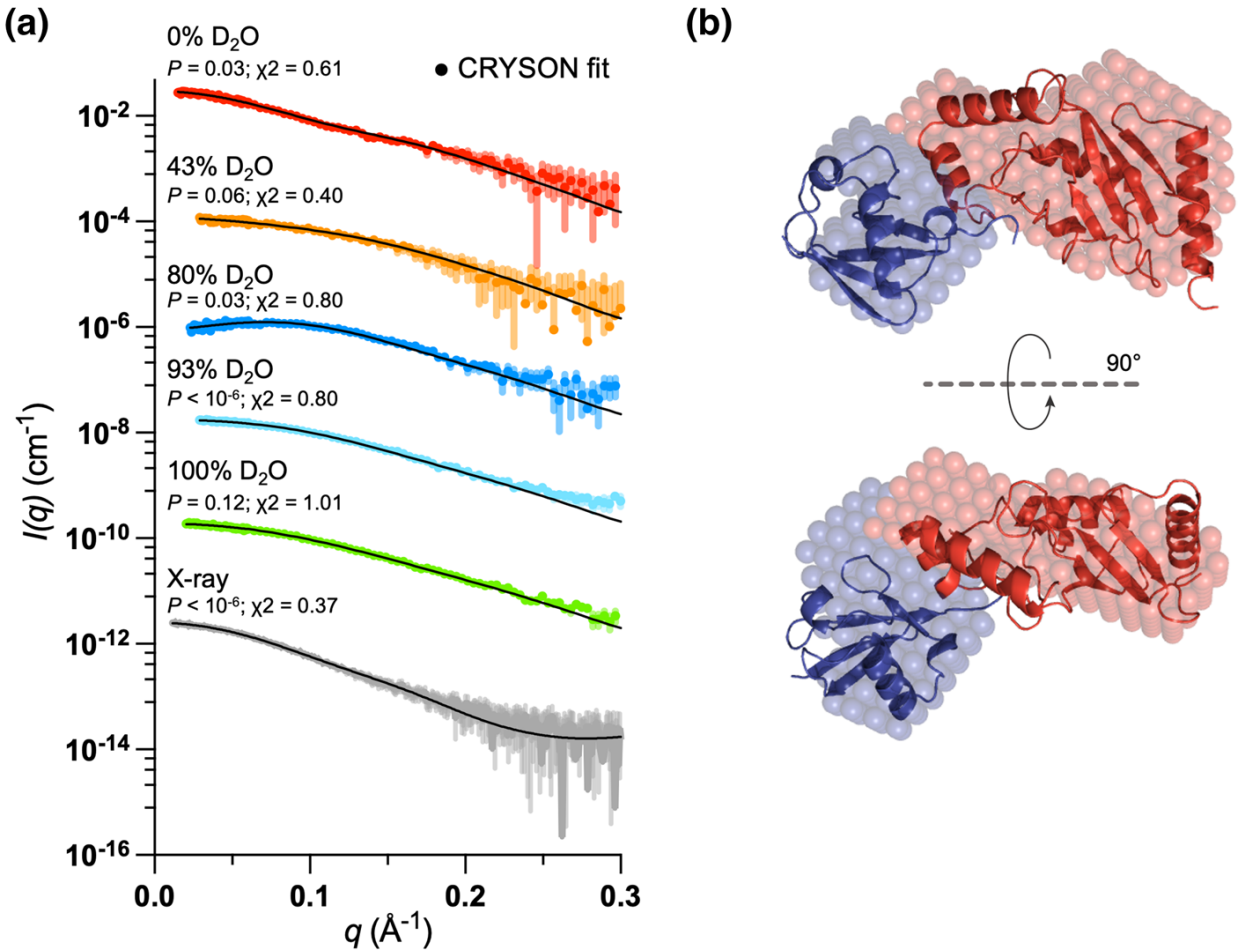
SAS scattering curves and models of hE2–dUb. **a** Background-subtracted scattering profiles at different D_2_O fractions, offset for clarity. The rigid-body model fits are represented as solid black lines. The match point of the hE2 is 43% D_2_O (orange) where dUb dominates the scattering. The match point of dUb is 100% D_2_O where hE2 is the dominant scatterer (green). **b** Superimposition of the ab initio (spheres) and rigid-body models. E2 is coloured red and Ub is in blue

SANS data were collected close to the hE2 “match-out” point at 43% D_2_O where dUb dominates the signal, at the dUb match-out point at 100% D_2_O where hE2 is the dominant scatterer, and at 0, 80, 93% D_2_O to obtain contrast variation data (Fig. 3A, Table S1). As described above and in Table S1, the SANS data were used to reconstruct a low-resolution multiphase model, shown in Fig. 3B. The derived ab initio model shows two well-defined domains within the system, a more compact, globular structure of Ub and a prolate shape of E2 (Fig. 3B). The crystal structures of Ub and E2 (PDB:4AP4) were used for rigid body modelling and the connection between the C-terminal Glycine of Ub and mutated Lysine 85 in hE2 was kept to 5 Å. The obtained rigid-body structure of hE2~dUb was fitted to SANS contrast variation series and SAXS data set. The final structure is presented in Fig. 3B and overlayed with the dummy-bead model. Both models show similar structural features of a highly extended conformation, suggesting a potential lack of noncovalent interactions between domains. The fits to the SAS data were assessed using CorMap showing a good agreement, except the 93% D_2_O dataset showing a discrepancy at higher angles and SAXS data with a poor fit in mid-range. The Stuhrmann analysis (Stuhrmann 1974) indicates that the molecule with the higher scattering length density must lay toward the periphery of the complex (Fig. S1) supporting the ab initio and high-resolution models showed in Fig. 3B. The consistency of the data suggests that produced modularly deuterated conjugate is highly stable is solution for over 44 h of measurement and Guinier analysis show no indication of aggregation at different fractions of D_2_O in the buffer, Fig. S2A.

### Flexibility of E2–Ub conjugate with isopeptide bond

**Fig. 4:**
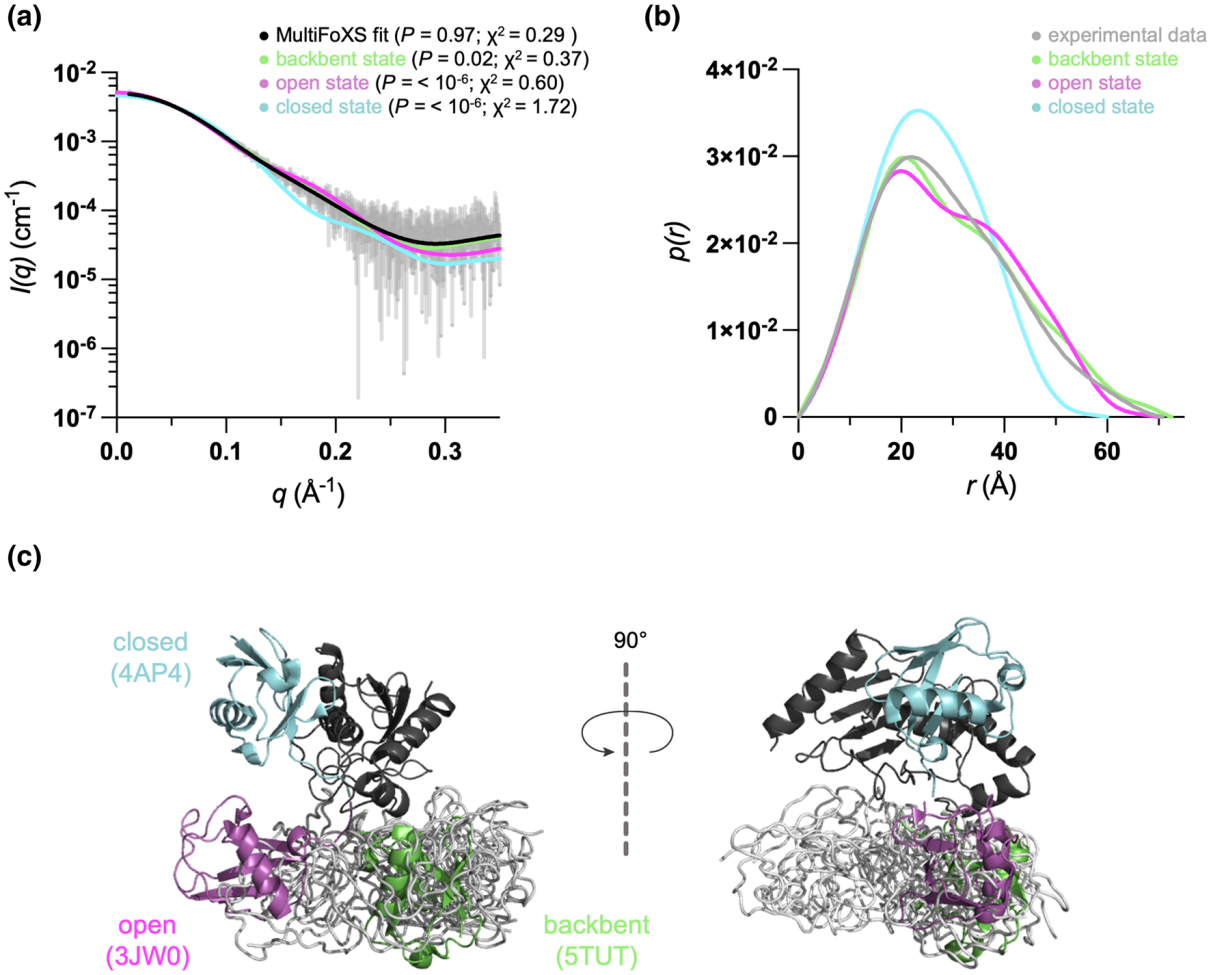
Evaluating flexibility of E2–Ub conjugate by multistate modelling. **a** Experimental SAXS data with calculated scattering intensities from the three E2–Ub conjugate states and a fit to the ensemble. **b** *p*(*r*) versus *r* profiles obtained from the data in B. **c** Superimposition of closed, open and backbent states with the ensemble. **d** The multistate ensemble indicating various conformations of E2–Ub, the sampled states mostly occupy the backbent conformation

The conformational landscape of E2–Ub conjugates is defined by the orientation of Ub within the complex with three distinct states (Page et al. 2012). The “closed” state, where Ub is placed against the crossover α2 helix of E2, is considered as an active state, as it allows transfer of Ub to the substrate by RING E3 ligase. In contrast, the “open” state Ub is located below the E2 active site, limiting contacts between the two proteins. In the “backbent” state, the conjugate forms the most extended conformation, with the Ub oriented close to the E2 loops 5 and 6 (Pruneda et al. 2011). The available crystal structures of open, closed and backbent states of UBE2D1 and homologs were assessed using CRYSOL and fitted to the experimental SAXS data (Fig. 4A). The computed solution intensities of the closed (PDB:4AP4) and open (3JW0) states display a poor fit to the experimental data, while a backbent UBE2D1~Ub structure (5TUT; highly similar to the UBE2D3~Ub structure 3UGB (Page et al. 2012)) reasonably fits the data (*p* value 0.2). In the Fig. 4B, the pairwise distance distribution profile, *p*(*r*)*,* derived from the SAXS data shows a single major peak with a shoulder. The *p*(*r*) profile of the open and backbent E2~Ub states illustrate a well-defined multimodularity of the conjugate with two distinguishable peaks and similar maximum particle dimension (*D*_max_ ~ 70–72 Å). In contrast, the *p*(*r*) analysis of the closed state indicates a well-folded compact molecule with a significantly smaller *D*_max_ value (around 60 Å). In Fig. 4C, the crystal structures are superimposed highlighting different orientations of Ub within the conjugate.

In the absence of E3 ligases, solution studies of the thioester and oxyester conjugated E2–Ub suggested that these complexes are highly dynamic and exhibit a range of conformations (Pruneda et al. 2011; Page et al. 2012). To further investigate the dynamics of E2–Ub with the isopeptide bond, we used MultiFoXS for multistate modelling to interpret the SAXS data, assuming the flexibility of Ub C-terminus residues 71–76 (Pruneda et al. 2011). The derived ensemble of ten structures provided an excellent fit to the SAXS data (*p* value 0.97). Ten obtained models of E2–Ub are superimposed showing various conformations, indicating significant flexibility of Ub in the complex. Interestingly, the sampled states mostly occupy the backbent conformation (Fig. 4C), where Ub is close to the C-terminal helix and E2 active site, with low tendency towards the open state. The closed state is not at all present in the ensemble in contrast to SAXS solution ensembles of UBE2D3~Ub and UBE2N~Ub (Pruneda et al. 2011).

In cells, E2s are mostly present as E2–Ub conjugates, ready to perform their functions (Siepmann et al. 2003). It has been speculated that E2 enzymes favour different relative orientations of Ub within the conjugate, and some can populate mostly the closed conformation, even in the absence of E3 ligases (Pruneda et al. 2011; Wickliffe et al. 2011). Available crystal structures of free E2s in the UBE2D family are both backbent (5TUT, 3UGB), and fit well with the current SANS-based model, suggesting that the backbent conformation is indeed predominant also in solution (Fig. 3A). However, the pronounced lack of closed-state representatives in the SAXS-based analysis (Fig. 4) is in contrast with early SAXS-derived ensembles of UBE2D3~Ub and UBE2N~Ub (Pruneda et al. 2011). This would suggest a possibility for single-point mutations in E2s at, or close to, the active site to also affect the distribution of interdomain orientations of E2–Ub conjugates in solution, which may affect the possibility for E3s to stabilise the active, closed state of the conjugate (Pruneda et al. 2012; Soss et al. 2013). A lower accessibility to the closed state in solution of the UBE2D1 mutant studied here (D87S) would plausibly be in agreement with its resistance to hydrolysis catalysed by TRIM21 (Anandapadamanaban et al. 2019). The current study shows the potential for SAS studies of a fuller range of UBE2D1 mutants conjugated to Ub to explore this further.

## Conclusions

The unique feature of SANS in investigating protein–protein complexes is the possibility to use solution contrast variation by adjusting H_2_O/D_2_O ratios and tailored protein deuteration levels. Generally, SAS lacks the high atomic resolution to resolve small individual components within modular complexes but allows studying the mechanism in solution and getting insight into protein flexibility and conformational changes. There are methods for the selective segmental labelling of protein domains using Sortase A catalysed ligation (Sonntag et al. 2017) or intein-mediated protein ligation (Wilkinson et al. 2005). However, the enzyme-mediated ligation requires an introduction of a recognition motif sequence, possibly altering the overall conformation. With the presented approach and data analysis, one can resolve the spatial disposition of proteins from E2–Ub conjugates in structural and dynamic detail. The future challenges include using this methodology to trap complexes of E2–Ub with different substrates, including E3 ligases. A recent SANS solution study shows ubistatin derivatives in complex with polyubiquitin connected by K48, K11 and K63 linkages (Nakasone et al. 2017). Modular deuteration of Ub within the different chains can help to resolve structural arrangements and provide another angle to understand the complexity of the molecular mechanisms also in larger complexes. Using established protein deuteration protocols we got large amounts of Ub, which are necessary for SANS experiments, as it requires a substantial amount of deuterated sample. The significant protein yield from 1 L of deuterated minimum media opens possibilities to test preparation of various labelled Ub-based probes used in studying ubiquitination pathways (Zhao et al. 2020).

## Supplementary Information

Below is the link to the electronic supplementary material.

Supplementary file1 (DOCX 209 KB)

Supplementary file2 (DOCX 51 KB)

## Data Availability

SAS data and the models are submitted to the Small Angle Scattering Biological Data Bank, ID: SASDP34.
