# Supplementary material for: Production and characterisation of modularly deuterated UBE2D1–Ub conjugate by small angle neutron and X-ray scattering
Source: Eur Biophys J. 2022 Oct 26;51(7-8):569–77. doi: 10.1007/s00249-022-01620-1 (PMC9675693; doi:10.1007/s00249-022-01620-1)
Supplement: Supplementary file 1 — Supplementary file1 (DOCX 209 KB) [file 249_2022_1620_MOESM1_ESM.docx]

**Supplementary Information**

Production and characterisation of modularly deuterated UBE2D1~Ub conjugate by Small Angle Neutron and X-ray Scattering

Zuzanna Pietras^a^, Anthony P. Duff^b^, Vivian Morad^a^, Kathleen Wood^c^, Cy M. Jeffries^d^, Maria Sunnerhagen^a,^*

^a^ Department of Physics, Chemistry and Biology, Division of Chemistry, Linköping University, 581 83 Linköping, Sweden

^b^ National Deuteration Facility, Australian Nuclear Science and Technology Organisation (ANSTO), New Illawarra Road, Lucas Heights, NSW 2234, Australia

^c^ Australian Nuclear Science and Technology Organisation (ANSTO), ﻿New Illawarra Road, Lucas Heights, NSW 2234, Australia

^d^ European Molecular Biology Laboratory (EMBL) Hamburg Outstation, 22607 Hamburg, ﻿Germany

* Corresponding author

E-mail address: [maria.sunnerhagen@liu.se](mailto:maria.sunnerhagen@liu.se)

**Figure S1. Stuhrmann analysis of hE2~dUb dataset.**

Stuhrmann plot of modularly deuterated hE2~dUb with the parabolic fit, where:

$R_{g}^{2}=R_{v}^{2}+ \frac{\alpha}{\rho^{-}}+ \frac{\beta}{\rho^{-2}}$.

Because the slope of alpha is positive, α >0, the component with a higher scattering density, dUb, is located further from the mass centre of the complex.

**Figure S2. Guinier plots for SAS data and predicted and experimental calculation of match point of the complex.**

**a.** Guinier plots of SAS data at all contrast points.

**b.** The relationship between the square root of forward scattering *I*(0) normalized by protein concentration and the fraction of D_2_O in the buffer is linear. Predicted match point of the whole complex is 63.3%. The match point based on linear regression from *I*(0) values extracted from Guinier approximation is 63.7% and from *p(r)* 64.2%. The experimental data is in the agreement with the predicted *I*(0) calculation and supports Guinier analysis, indicating that the samples were monodisperse and pure.

**Supplementary Table 1** includes information about: data acquisition and reduction, detailed information about protein samples, data analysis of structural parameters and validation, modelling approach and validation.
