# Supplementary material for: Production and characterisation of modularly deuterated UBE2D1–Ub conjugate by small angle neutron and X-ray scattering
Source: Eur Biophys J. 2022 Oct 26;51(7-8):569–77. doi: 10.1007/s00249-022-01620-1 (PMC9675693; doi:10.1007/s00249-022-01620-1)
Supplement: Supplementary file 2 — Supplementary file2 (DOCX 51 KB) [file 249_2022_1620_MOESM2_ESM.docx]

| **Sample details** | |
| --- | --- |
| Organism | Homo sapiens (Human) |
| UniProt ID | UBE2D1: P51668 (1-147)  Ub: P0CG48 (1-76) |
| Sequence | GAGSGSMALKRIQKELSDLQRDPPAHCRAGPVGDDLFHWQATIMGPPDSAYQGGVFFLTVHFPTDYPFKPPKIAFTTKIYHPNINSNGSIKLDILRSQWSPALTVSKVLLSICSLLCDPNPDDPLVPDIAQIYKSDKEKYNRHAREWTQKYAM (UBE2D1);  GSHMQIFVKTLTGKTITLEVEPSDTIENVKAKIQDKEGIPPDQQRLIFAGKQLEDGRTLSDYNIQKESTLHLVLRLRGG (Ubiquitin) |
| Extinction coefficient  (A280 nm, 0.1% w/v) | 1.496 (UBE2D1);  0.168 (Ubiquitin);  1.043 (UBE2D1-Ub) |
| Molecular mass  (from sequence) (kDa) | 25,94 |
| Partial specific volume  (cm^3^ g^-1^) | 0.738 |

| Sample at v/v % D_2_O | hE2~dUb  0% D_2_O | hE2~dUb  43% D_2_O | hE2~dUb  80% D_2_O | hE2~dUb  93% D_2_O | hE2~dUb  100% D_2_O | hE2~dUb  (SAXS) |
| --- | --- | --- | --- | --- | --- | --- |
| ﻿Contrast, Δρ (10^10^ cm ^-2^) | 3.554 | 1.14127 | -0.935 | -1.66423 | -2.057 | 2.825 |
| Protein concentration (mg ml^-1^) | 4.02 | 3.92 | 3.78 | 3.70 | 3.65 | 3.78 |

| **Data collection parameters** | | |
| --- | --- | --- |
|  | SANS | SAXS |
| Instrument | ﻿QUOKKA (ANSTO) | ﻿SAXS-WAXS (Australian Synchrotron) |
| Wavelength (Å) | 6.00 | ﻿1.078 |
| Beam geometry | Point | Point |
| Sample-to-detector distance (m) | ﻿1.3 (short); 6.0 (long) | ﻿2.5 |
| *q*-range (Å^-1^) | 0.03-0.40 (short); 0.01-0.09 (long) | 0.01 - 0.33 |
| Exposure time (s) | SDD 6m: 3,600 (0% D_2_O); 45,000 (43%); 21,600 (80%); 45,000 (93%); 3,600 (100%)  SDD 1.3m: 3,600 (0% D_2_O); 10,800 (43%); 10,800 (80%); 10,800 (93%); 3,600 (100%) | 1x423 frames |
| Measurement type | Batch; Neutron contrast variation.  Hellma 120-QS ﻿1.0-mm quartz cell | SEC-SAXS, Superdex Inc 5/150 column (GE Healthcare) at flow rate 0.2 mL min^-1^;  1.0-mm quartz capillary |
| Temperature (°C) | 10 | 27 |
| Absolute intensity calibration | Incident beam intensity | Water |
| **Data reduction, analysis, and modelling** | | |
| SANS data reduction | | IgorPro |
| SAXS data reduction | | ScatterBrain |
| Extinction coefficient | | ProtParam (Gasteiger et al. 2005) |
| Calculation of volume and contrast | | MULCh 1.1 |
| Structural parameters analysis | | Primus (ATSAS 3.0.1) |
| Stuhrmann analysis | | MULCh 1.1 |
| Bead modelling | | MONSA, (ATSAS online) |
| Atomic structure modelling | | SASREFCV (ATSAS 3.0.1) |
| Three-dimensional graphic model representation | | PyMOL 2.0.6 |

| **Structural parameter analysis** | | | | | | |
| --- | --- | --- | --- | --- | --- | --- |
| Sample | hE2~dUb  0% D_2_O | hE2~dUb  43% D_2_O | hE2~dUb  80% D_2_O | hE2~dUb  93% D_2_O | hE2~dUb  100% D_2_O | hE2~dUb  (SAXS) |
| ***Guinier analysis*** | | | | | | |
| *I*(0) (cm^-1^) | 0.0298  (± 0.0003) * | 0.0028  (± 0.00008) * | 0.0016  (± 0.00004) * | 0.0062  (± 0.00003) * | 0.0099  (± 0.00005) *^[[1]](#footnote-1)^ | 0.0049  (± 0.00004) |
| R_g_ (Å) | 21.7 (± 0.3) | 12.0 (± 0.6) | -17.9 (± 0.2) | 13.2 (± 0.2) | 14.9 (± 0.1) | 21.6 (± 0.3) |
| ***P(r) analysis*** | | | | | | |
| *I*(0) (cm^-1^) | 0.0296  (± 0.2) * | 0.0029  (± 0.2) * | N/A | 0.0061  (± 0.2) * | 0.0100  (± 0.2) * | 0.0050  (± 0.00004) |
| R_g_ (Å) | 22.5 (± 0.2) | 12.1 (± 0.2) | N/A | 12.3 (± 0.2) | 15.4 (± 0.2) | 21.8 (± 0.2) |
| D_max_ (Å) | 74 | 32 | 79 | 76 | 53 | 71 |
| Total estimate from GNOM | 0.81 | 0.87 | 0.50 | 0.98 | 0.76 | 0.89 |
| Quality of fit χ^2^/CorMap *P-value* | 0.54/0.65 | 0.52/0.90 | 0.84/0.41 | 0.60/0.06 | 0.90/0.13 | 0.30/0.99 |
| Porod volume (10^3^Å^3^) | N/A | N/A | N/A | N/A | N/A | 34 |
| ***Molecular weight determination*** | | | | | | |
| MW *I*(0) (kDa) | 26.1 | 23.0 | 20.4 | 26.3 | 25.8 | N/A |
| MW Vc (kDa) (Rambo and Tainer 2013) | N/A | N/A | N/A | N/A | N/A | 24.2 |
| MW SAXSMoW  (kDa) (Fischer et al. 2010) | N/A | N/A | N/A | N/A | N/A | 25.7 |
| **Shape model-fitting results** | | | | | | |
| ***MONSA*** | | | | | | |
| q-range for fitting | 0.015 – 3.66 | 0.029 – 3.72 | 0.023 – 3.79 | 0.029 – 3.76 | 0.020 – 3.83 | N/A |
| Quality of fit χ^2^/CorMap *P-value* | 0.59/0.00 | 0.42/0.00 | 1.03/0.00 | 1.11/0.00 | 1.08/0.00 | N/A |
| Symmetry | P1 | | | | | N/A |
| ***SASREFCV*** | | | | | | |
| Starting crystal structure | PDB: A4P4 | | | | | |
| Quality of fit χ^2^/CorMap *P-value* | 0.61/0.03 | 0.40/0.06 | 0.80/0.03 | 0.80/0.00 | 1.01/0.12 | 0.37/0.00 |
| **Multistate models** *MultiFoXS (10 000 models in starting set)* | | | | | | |
| Starting crystal structure | A4P4 | | | | | |
| Flexible residues | 71-76 (C-term of Ubiquitin) | | | | | |
| Quality of fit χ^2^/CorMap *P-value* | 0.29/0.97 | | | | | |
| Number of states | 2 | | | | | |

**References**

Fischer H, Neto M de O, Napolitano HB, et al (2010) Determination of the molecular weight of proteins in solution from a single small-angle X-ray scattering measurement on a relative scale. J Appl Crystallogr 43:101–109. https://doi.org/10.1107/s0021889809043076

Gasteiger E, Hoogland C, Gattiker A, et al (2005) The Proteomics Protocols Handbook. 571–607. https://doi.org/10.1385/1-59259-890-0:571

Rambo RP, Tainer JA (2013) Accurate assessment of mass, models and resolution by small-angle scattering. Nature 496:477–481. https://doi.org/10.1038/nature12070

1. * The stated *I*(0) (cm^-1^) values for the SANS data are from the concentration-normalised data. [↑](#footnote-ref-1)
